# Comparative effectiveness of GnRH antagonist, mild stimulation, and PPOS protocols in POSEIDON groups 3 and 4: a retrospective cohort study with entropy balancing

**DOI:** 10.3389/fendo.2026.1969038

**Published:** 2026-10-21

**Authors:** Haiyue Zhao, Yunping Ni, Aiai Wang, Juan Liu, Xinghong Lu, Shan Lu

**Affiliations:** Department of Reproductive Medicine, Guangdong Provincial Hospital of Chinese Medicine, The Second Affiliated Hospital of Guangzhou University of Chinese Medicine, Guangzhou, China

**Keywords:** cumulative live birth rate, diminished ovarian reserve, entropy balancing, GnRH antagonist, mild stimulation, poor ovarian response, POSEIDON criteria, progestin-primed ovarian stimulation

## Abstract

**Background:**

Patients classified as POSEIDON groups 3 and 4 have diminished ovarian reserve and predicted poor ovarian response. Although GnRH antagonist, mild stimulation, and progestin-primed ovarian stimulation (PPOS) protocols are widely used, direct comparative evidence from homogeneous cohorts remains limited.

**Methods:**

This retrospective cohort study included 592 first IVF cycles (GnRH antagonist: n = 203, mild stimulation: n = 243, PPOS: n = 146) in patients whose sole IVF indication was POSEIDON groups 3 or 4. Entropy balancing achieved near-perfect balance across nine baseline covariates. The primary outcome was cumulative live birth rate per initiated cycle.

**Results:**

After entropy balancing, cumulative live birth rates showed no statistically significant differences: 32.8% for antagonist, 29.2% for mild, and 35.8% for PPOS (global *P* = 0.452). Cumulative clinical pregnancy rates were also similar (37.9%, 34.6%, and 39.5%; *P* = 0.649). Weighted logistic regression confirmed no significant protocol differences (all *P* > 0.05). No significant interaction was observed between protocol and POSEIDON subgroup for either outcome (*P* for interaction = 0.384 and 0.213). Although the antagonist protocol yielded superior embryological outcomes (more oocytes, usable embryos, and high-quality embryos, all *P* < 0.001), this did not translate into higher cumulative live birth or pregnancy rates. Critically, rates of no oocytes retrieved (6.0%, 6.4%, and 7.6%; *P* = 0.888) and cancellation due to no usable embryos (16.5%, 19.3%, and 16.2%; *P* = 0.786) were nearly identical across groups, suggesting a potential threshold effect that warrants further investigation, as protocol choice did not significantly affect the probability of obtaining at least one embryo in this exploratory analysis. PPOS had the lowest premature LH surge rate (1.5%, global *P* = 0.047).

**Conclusions:**

In POSEIDON groups 3 and 4 patients, the three protocols showed no statistically significant differences in cumulative live birth and clinical pregnancy rates despite differences in embryo yield. This exploratory finding suggests that embryo obtainability may be more strongly associated with intrinsic patient characteristics than with protocol choice, although this requires confirmation in future prospective studies.

## 1 Introduction

Diminished ovarian reserve (DOR) presents one of the most formidable challenges in assisted reproductive technology (ART), fundamentally characterized by a progressive decline in both oocyte quantity and quality. This condition, typically indicated by reduced antral follicle count (AFC), low anti-Müllerian hormone (AMH) levels, and elevated basal follicle-stimulating hormone (FSH) (1), affects a substantial proportion of women seeking fertility treatment. The clinical consequence—poor ovarian response (POR)—occurs in approximately 9–24% of women undergoing ovarian stimulation for *in vitro* fertilization (IVF) (2), and remains a primary concern for reproductive specialists due to its strong association with reduced oocyte yield, lower euploidy rates, diminished pregnancy outcomes, and increased cycle cancellation (3, 4).

The evolution of POR classification reflects our growing understanding of its heterogeneous nature. The Bologna criteria, introduced in 2011, represented the first international consensus attempt to standardize POR diagnosis (5). However, these criteria have been subject to debate regarding their heterogeneity and clinical applicability (6, 7). The Patient-Oriented Strategies Encompassing Individualized Oocyte Number (POSEIDON) criteria, introduced in 2016, marked a paradigm shift by integrating key biomarkers—age, AFC, and AMH—to create a more nuanced, biologically coherent classification of low-prognosis patients (8). This refined stratification enables more personalized therapeutic approaches by distinguishing four distinct subgroups with different expected outcomes (9–11). Our study focuses specifically on POSEIDON Group 3 (women < 35 years) and Group 4 (women ≥ 35 years), both defined by AFC < 5 and/or AMH < 1.2 ng/mL—a population of anticipated poor responders who pose particularly demanding clinical management challenges.

Several ovarian stimulation protocols are commonly employed in these patients. The GnRH antagonist protocol remains widely used for its effective suppression of premature luteinizing hormone (LH) surges, cycle flexibility, and favorable safety profile (12). Mild stimulation protocols, employing clomiphene citrate or letrozole with low-dose gonadotropins, are valued for their reduced medication burden, lower cost, and patient-friendly approach (13, 14). More recently, progestin-primed ovarian stimulation (PPOS) has emerged as a promising alternative, utilizing oral progestins to block the LH surge while eliminating the need for injectable GnRH analogs and maintaining competitive embryological outcomes (15–18).

Despite these advances, the existing literature comparing these protocols in POR patients has largely been limited to pairwise comparisons—PPOS versus GnRH antagonist (16, 19, 20), GnRH antagonist versus mild stimulation (21, 22), and mild stimulation versus PPOS (23, 24)—with few studies providing a comprehensive, head-to-head evaluation within a homogeneous POSEIDON-stratified cohort. A recent conference abstract reported a three-way comparison of these protocols (25), but that study examined blastocyst yield in women aged 21–35 years rather than cumulative live birth in POSEIDON 3/4 populations, leaving the comparative effectiveness for cumulative live birth in this specific population incompletely characterized. Moreover, many comparative studies have included heterogeneous patient populations with mixed infertility etiologies, such as tubal factors, male factors, or endometriosis (19, 20). This heterogeneity may confound the interpretation of protocol-specific effects in the POR population, as the impact of these other etiologies on outcomes may obscure the intrinsic differences between stimulation protocols.

For patients with POR, the cumulative live birth rate (CLBR)—defined as the probability of achieving at least one live birth from all fresh and frozen embryos derived from a single stimulation cycle—is a particularly meaningful endpoint (26). Unlike per-transfer outcomes, CLBR accounts for the reality that poor-prognosis patients often require multiple embryo transfers to achieve a live birth, and it reflects the overall efficiency of a stimulation protocol in maximizing the cumulative likelihood of achieving at least one live birth. However, comparative CLBR data in rigorously selected POSEIDON 3/4 populations remain scarce.

To provide additional evidence on this clinically important question, we conducted a retrospective cohort study with several key methodological features. First, we restricted inclusion to women undergoing their first IVF cycle, which eliminates the confounding influence of prior cycle outcomes on protocol selection—a critical advantage that ensures protocol assignment is not influenced by previous treatment results. Second, we applied rigorous patient selection criteria, restricting to women whose sole indication for IVF was meeting POSEIDON Group 3 or 4 criteria, minimizing confounding from other infertility etiologies. Third, we performed a head-to-head comparison of all three protocols within a uniformly stratified population. Fourth—and most importantly from a methodological standpoint—we employed entropy balancing (27), a state-of-the-art weighting method that achieves exact covariate balance while retaining the full analytical sample, offering a distinct advantage over propensity score-based approaches that inevitably discard unmatched observations. This method, despite being well-established in econometrics and causal inference, has rarely been applied in reproductive medicine, and its adoption here represents a methodological advancement for comparative effectiveness research in this field. We evaluated a comprehensive range of outcomes, including CLBR, cumulative clinical pregnancy rate (CCPR), embryological parameters, and cycle characteristics, to provide robust evidence for clinical decision-making in this challenging population.

## 2 Materials and methods

### 2.1 Study design and ethical approval

This single-center, retrospective cohort study was conducted at the Reproductive Medicine Center of Guangdong Provincial Hospital of Traditional Chinese Medicine. The study protocol was reviewed and approved by the Medical Ethics Committee of Guangdong Provincial Hospital of Traditional Chinese Medicine (Approval No. ZE2025-354-01). All procedures were performed in accordance with the principles of the Declaration of Helsinki. The committee waived the requirement for written informed consent due to the retrospective design and the use of anonymized data.

### 2.2 Study population and eligibility criteria

The study screened all first IVF cycles performed between January 2017 and June 2023. Inclusion was restricted to women whose sole indication for IVF was fulfilling the POSEIDON criteria for diminished ovarian reserve: Group 3 (age < 35 years; AFC < 5 and/or AMH < 1.2 ng/mL) or Group 4 (age ≥ 35 years; AFC < 5 and/or AMH < 1.2 ng/mL). Only cycles utilizing one of the three ovarian stimulation protocols of interest—GnRH antagonist, mild stimulation, or PPOS—were included.

To establish a homogeneous cohort and minimize confounding, we applied the following strict exclusion criteria: (1) uterine or tubal pathology, including untreated hydrosalpinx, moderate-to-severe endometriosis (ASRM stage III/IV), adenomyosis, submucous or intramural fibroids ≥ 4 cm in diameter, and congenital uterine anomalies; (2) endocrine disorders, including polycystic ovary syndrome, hyperprolactinemia, or uncontrolled thyroid dysfunction; (3) chronic diseases known to affect gonadal function or pregnancy outcomes (e.g., tuberculosis); (4) known karyotypic abnormalities in either partner; (5) severe male factor infertility requiring intracytoplasmic sperm injection (ICSI); and (6) cycles with missing essential data.

### 2.3 Data collection

Comprehensive demographic, clinical, and laboratory data were extracted from electronic medical records. Collected variables included: patient age; body mass index (BMI); AFC; AMH; baseline FSH, LH, and estradiol (E_2_); infertility type and duration; total gonadotropin (Gn) dose; Gn stimulation duration; starting Gn dose; trigger-day LH, E_2_, and progesterone (P); number of total (≥ 10 mm) and dominant (≥ 14 mm) follicles; oocytes retrieved; metaphase II (MII) oocytes; two-pronuclei (2PN) zygotes; usable embryos; and high-quality embryos. We also collected data on clinical pregnancy and live birth outcomes for all embryo transfers derived from the initiated cycle, which formed the basis for calculating cumulative clinical pregnancy rate and cumulative live birth rate per initiated cycle.

### 2.4 Ovarian stimulation protocols

Ovarian stimulation was initiated on cycle day 2 or 3 after a baseline ultrasound scan. Patients underwent one of three protocols: (1) PPOS protocol: Daily oral medroxyprogesterone acetate (8–10 mg/day) with human menopausal gonadotropin (HMG; 150–300 IU/day) until trigger. (2) Mild stimulation protocol: Clomiphene citrate (50–100 mg/day) or letrozole (2.5–5 mg/day) with low-dose HMG (75–150 IU/day) until trigger. In the mild stimulation protocol, no additional LH-suppressing agents (such as GnRH antagonists or progestins) were administered. (3) GnRH antagonist protocol: Recombinant FSH (rFSH; 150–300 IU/day) or HMG (150–300 IU/day) with cetrorelix (0.25 mg/day) initiated when the leading follicle reached 12–14 mm or serum LH exceeded a predetermined threshold.

Gn dosing was individualized and adjusted every 2–3 days based on transvaginal ultrasound monitoring of follicular growth and serum E_2_. Final oocyte maturation was triggered with urinary hCG (8000–10,000 IU), recombinant hCG (250 μg), or triptorelin acetate (0.1 mg) when at least one follicle reached 18 mm, two follicles reached 17 mm, or three follicles reached 16 mm. Transvaginal ultrasound-guided oocyte retrieval was performed 33–36 hours after trigger.

### 2.5 Embryo culture and assessment

Oocyte-cumulus complexes were cultured in single-step medium under mineral oil. Insemination was performed 4–6 hours post-retrieval via IVF; ICSI was excluded. Fertilization was assessed 16–18 hours post-insemination by identification of two distinct pronuclei. Normally fertilized zygotes were individually cultured in a tri-gas incubator at 37 °C, 6% CO_2_, 5% O_2_.

Usable embryos were defined as cleavage-stage embryos with 6–10 blastomeres, moderate cell symmetry, fragmentation ≤ 25%, and no multinucleation (corresponding to Cummins grades I–III) (28), or blastocysts with expansion grade ≥ 3 and inner cell mass and trophectoderm grades of at least C (corresponding to Gardner grade ≥ 3CC) (29), which were deemed suitable for fresh transfer or vitrification. High-quality embryos were defined more stringently as cleavage-stage embryos with exactly 8 blastomeres, even-sized and symmetrical blastomeres, fragmentation ≤ 10%, and no multinucleation (corresponding to Cummins grade I) (28), or blastocysts graded ≥ 3BB according to the Gardner and Schoolcraft system (29).

### 2.6 Embryo transfer and luteal support

Cycle cancellation criteria included: (1) failure to obtain any oocytes; (2) absence of usable embryos after fertilization and culture; (3) premature LH surge (serum LH > 15 mIU/mL on or before the day of trigger); (4) endometrial thickness < 8 mm on the scheduled transfer day or unfavorable endometrial morphology; (5) elevated serum progesterone level (≥ 4.77 nmol/L, equivalent to 1.5 ng/mL) on the day of trigger; or (6) risk of moderate-to-severe ovarian hyperstimulation syndrome.

Fresh Embryo Transfer: In the GnRH antagonist group, fresh embryo transfer was performed on day 3 or day 5 provided that endometrial conditions were favorable (thickness ≥ 8 mm and acceptable morphology) and no contraindications (e.g., elevated progesterone level or risk of ovarian hyperstimulation syndrome) were present. If these criteria were not met, embryo transfer was deferred, and all viable embryos were vitrified for subsequent frozen-thawed embryo transfer (FET).

Frozen-thawed Embryo Transfer: FET was performed in patients who underwent a freeze-all strategy, including all patients in the PPOS and mild stimulation groups, and those in the GnRH antagonist group who did not meet the criteria for fresh embryo transfer. FET was performed using individualized endometrial preparation protocols based on the patient’s ovulatory status. (1) Natural Cycle Protocol: For women with regular ovulation. Vaginal progesterone gel (90 mg/day) was initiated on the day of confirmed ovulation by ultrasonography. Cleavage-stage embryos or blastocysts were transferred 3 or 5 days later, respectively. (2) Artificial Cycle Protocol: For patients with irregular menstruation. Oral estradiol valerate (2–8 mg/day) was administered starting on day 3 of the menstrual cycle. After 12–14 days, if endometrial thickness reached ≥ 8 mm, oral micronized progesterone (200–400 mg/day) was added. Embryo transfer was performed on the third day of progesterone administration for cleavage-stage embryos and on the fifth day for blastocysts. (3) Ovulation Induction Cycle Protocol: For anovulatory patients. Letrozole (2.5–5 mg/day) was administered for 5 days starting from cycle day 2-3. If the response was suboptimal, low-dose gonadotropin (HMG; 75–150 IU/day) was added sequentially. Final follicle maturation was triggered with r-hCG (250 μg) when the leading follicle reached ≥ 18 mm in diameter.

Luteal Support: Luteal phase support was provided from the day of oocyte retrieval (fresh cycles) or progesterone initiation (FET cycles) using vaginal progesterone gel (90 mg/day) combined with oral dydrogesterone (10–20 mg/day). Medications were continued until the pregnancy test 14 days after embryo transfer. Upon confirmed clinical pregnancy, support was tapered starting from gestational week 8 and discontinued by week 10.

### 2.7 Outcome measures

Live birth was defined as the delivery of at least one live-born infant after 28 completed weeks of gestation. The counting unit for all live birth outcomes in this study was per initiated cycle. Cumulative live birth rate (CLBR) was defined as the probability of achieving at least one live birth from all fresh and/or frozen embryo transfers derived from a single stimulation cycle, continuing until a live birth occurred or all embryos were used. A conservative assumption was adopted: couples who discontinued treatment were ascribed a zero probability of subsequent conception. The follow-up period for this outcome was set at two years after oocyte retrieval. The denominator for CLBR was per initiated cycle.

Cumulative clinical pregnancy rate (CCPR) was defined as the probability of achieving at least one clinical pregnancy (defined as ultrasonographic visualization of at least one gestational sac) from all fresh and/or frozen embryo transfers derived from a single stimulation cycle. A conservative assumption was adopted: couples who discontinued treatment were ascribed a zero probability of subsequent conception. The follow-up period for this outcome was set at two years after oocyte retrieval. The denominator for CCPR was per initiated cycle.

Secondary outcomes included ovarian response parameters (total and dominant follicles), embryological outcomes (oocytes retrieved, MII oocytes, 2PN zygotes, usable and high-quality embryos), and key cycle events (cycles with no oocytes retrieved, cancellation due to no usable embryos, and premature LH surge rate [serum LH > 15 mIU/mL on or before the day of trigger]).

### 2.8 Statistical analysis

All statistical analyses were conducted using R software (version 4.5.1). Categorical variables are presented as frequencies and percentages. Continuous variables are presented as medians with interquartile ranges (IQR) given their non-normal distributions, as confirmed by Shapiro-Wilk tests. For unweighted comparisons, group differences were assessed using Kruskal-Wallis tests for continuous variables and chi-square tests for categorical variables. To address the inherent baseline imbalances typical of retrospective cohort studies, which can confound comparisons of treatment outcomes, we employed entropy balancing (27) using the ebal method implemented in the WeightIt package in R. Entropy balancing was performed to achieve covariate balance across the three treatment groups simultaneously. Weights were calibrated so that the weighted distributions of all prespecified covariates were balanced among the GnRH antagonist, mild stimulation, and PPOS groups. The optimization procedure achieves exact balance on the prespecified covariates, including balance on the first and, where applicable, second moments of the covariate distributions, thereby achieving more comprehensive covariate adjustment than methods that rely solely on mean balance. The entropy distance between the weight distribution and a uniform distribution is minimized, which ensures that the weights remain as close to equal as possible, minimizing the loss of effective sample size while achieving covariate balance.

Compared with propensity score matching, which discards untreated observations that cannot be matched and often reduces sample size, entropy balancing retains the full analytical sample. Compared with inverse probability of treatment weighting, which relies on correctly specified propensity score models and may produce unstable weights due to extreme propensities, entropy balancing directly optimizes covariate balance without requiring a model for treatment assignment, thereby avoiding the risk of model misspecification. Furthermore, entropy balancing naturally accommodates multi-group comparisons (three groups in our study) by simultaneously satisfying balance constraints across all groups, whereas propensity score methods are typically designed for two-group comparisons.

Entropy balancing was implemented using the ebal method in the WeightIt package (version 1.7.0) in R (version 4.5.1), with the average treatment effect (ATE) as the estimand. Only first moments were balanced (moments = 1); no quantile constraints or interaction terms were included. The optimization algorithm converged using the default tolerance level (maximum absolute mean difference < 1e-8). Among all covariates included in the entropy balancing procedure, only AMH had missing values (6/592, approximately 1.0%). Missing AMH values were imputed using predictive mean matching. All other covariates were complete. Weights were not truncated. Based on clinical relevance and prior literature, nine covariates were prespecified for entropy balancing: age, AMH, AFC, baseline FSH, baseline LH, baseline E_2_, BMI, infertility duration, and infertility type. To mitigate the influence of extreme values and improve balance on the distributional tails, baseline LH and baseline E_2_ were log-transformed (log[LH + 1] and log[E_2_+1]) prior to entropy balancing. POSEIDON group was not included as a covariate, as it is deterministically derived from age, AMH, and AFC; its inclusion would introduce perfect multicollinearity and compromise the entropy balancing algorithm. After weighting, all nine prespecified covariates achieved near-perfect balance with SMD below 0.001 (**Figure 1**). Weight diagnostics, including effective sample sizes, are reported in **Supplementary Table 1**. Balance was assessed using standardized mean differences (SMD), defined for multi-group comparisons as the maximum absolute difference between any two groups. An SMD < 0.1 was prespecified as the threshold indicating good balance.

**Figure 1:**
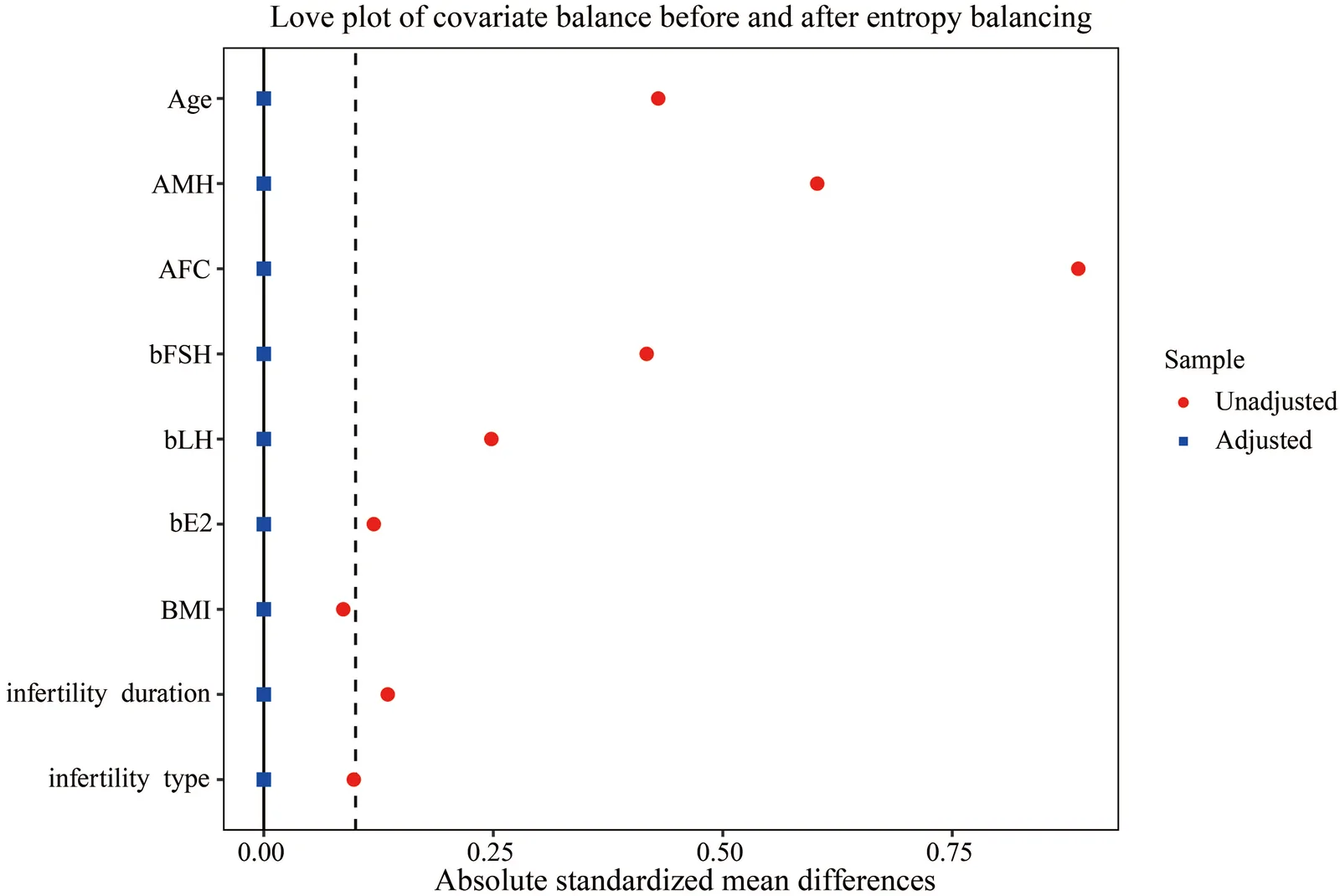
Love plot of covariate balance before and after entropy balancing. Covariate balance before (red circles) and after (blue squares) entropy balancing is compared. The x-axis represents the standardized mean difference (SMD), and the y-axis lists the covariates. The vertical dashed line at SMD = 0.1 indicates the prespecified threshold for good balance. SMD was calculated as the maximum absolute difference between any two of the three groups.

After entropy balancing, all subsequent analyses were performed using the derived weights. Continuous variables are presented as weighted medians with weighted IQR; group comparisons used weighted Kruskal-Wallis tests. Categorical variables are presented as weighted percentages; group comparisons used weighted chi-square tests. Weighted proportions with 95% confidence intervals (CI) were estimated using the Wilson method with continuity correction.

Holm adjustment was applied to all pairwise comparisons for the two primary outcomes (CLBR and CCPR) as well as for the secondary embryological outcomes reported in **Table 1**, to control the family-wise error rate at α = 0.05. For the multivariable logistic regression models (**Table 2**), the pairwise contrasts were derived from a single model. These are not independent tests but rather different parameterizations of the same model. Therefore, we did not apply additional multiplicity correction beyond the model-based Wald tests, as these contrasts are derived from the same model and represent different parameterizations rather than independent hypotheses. The exploratory subgroup analyses (**Supplementary Table 2**) were interpreted with caution given the limited power for interaction tests, and the significance threshold was set at α = 0.10 as prespecified in the table notes.

**Table 1:** Weighted ovarian stimulation and embryological outcomes per initiated cycle.

Variable	GnRH antagonist (n= 203)	Mild stimulation (n = 243)	PPOS (n = 146)	*P* value*	Holm-adjusted *P* values†
Gn starting dose (IU)	225 (150–300)	150 (150–180)	225 (225–300)	<0.001	Ant vs Mild: <0.001; Ant vs PPOS: 0.024; Mild vs PPOS: <0.001
Gn total dosage (IU)	2089 (1575–2746)	1400 (1200–1650)	2700 (2025–3000)	<0.001	Ant vs Mild: <0.001; Ant vs PPOS: 0.020; Mild vs PPOS: <0.001
Gn duration (days)	10 (9–12)	10 (8–11)	11 (9–13)	<0.001	Ant vs Mild: <0.001; Ant vs PPOS: 0.344; Mild vs PPOS: <0.001
Total follicles (≥10 mm), n	6 (2–8)	4 (2–5)	4 (2–7)	<0.001	Ant vs Mild: <0.001; Ant vs PPOS: <0.001; Mild vs PPOS: 0.005
Dominant follicles (≥14 mm), n	4 (2–6)	3 (1–4)	3 (2–5)	<0.001	Ant vs Mild: <0.001; Ant vs PPOS: <0.001; Mild vs PPOS: 0.004
Oocytes retrieved, n	4 (2–7)	3 (1–4)	4 (1–5)	<0.001	Ant vs Mild: <0.001; Ant vs PPOS: <0.001; Mild vs PPOS: 0.019
MII oocytes, n	3 (2–6)	2 (1–4)	3 (1–4)	<0.001	Ant vs Mild: <0.001; Ant vs PPOS: <0.001; Mild vs PPOS: 0.027
LH (U/L)	3.3 (2.1–6.6)	6.3 (3.3–9.9)	3.3 (2.0–6.0)	<0.001	Ant vs Mild: <0.001; Ant vs PPOS: 0.068; Mild vs PPOS: <0.001
E_2_ (pmol/L)	4108 (2394–6177)	3919 (2117–6689)	2941 (1391–5180)	<0.001	Ant vs Mild: <0.001; Ant vs PPOS: <0.001; Mild vs PPOS: 0.218
Progesterone (nmol/L)	1.6 (0.7–2.9)	1.1 (0.7–1.6)	1.9 (0.8–4.1)	<0.001	Ant vs Mild: <0.001; Ant vs PPOS: 0.912; Mild vs PPOS: <0.001
2PN zygotes, n	3 (1–5)	2 (1–3)	2 (1–4)	<0.001	Ant vs Mild: <0.001; Ant vs PPOS: <0.001; Mild vs PPOS: 0.015
Usable embryos, n	2 (1–4)	1 (1–3)	2 (1–3)	<0.001	Ant vs Mild: <0.001; Ant vs PPOS: <0.001; Mild vs PPOS: 0.007
High-quality embryos, n	1 (0–2)	0 (0–1)	0 (0–1)	<0.001	Ant vs Mild: <0.001; Ant vs PPOS: <0.001; Mild vs PPOS: 0.150
Cycles with no oocytes retrieved, %	6.0	6.4	7.6	0.888	Ant vs Mild: 1.000; Ant vs PPOS: 1.000; Mild vs PPOS: 1.000
Cycle cancellation due to no usable embryos, %	16.5	19.3	16.2	0.786	Ant vs Mild: 1.000; Ant vs PPOS: 1.000; Mild vs PPOS: 1.000
Premature LH surge, %	4.1	8.6	1.5	0.047	Ant vs Mild: 0.470; Ant vs PPOS: 0.470; Mild vs PPOS: 0.005

Data are weighted estimates after entropy balancing.

Continuous variables are presented as weighted median (weighted interquartile range). Categorical variables are presented as weighted percentages.

Total follicles were defined as the number of follicles ≥10 mm in diameter on the trigger day; dominant follicles were defined as the number of follicles ≥14 mm. 2PN zygotes refer to normally fertilized oocytes with two distinct pronuclei observed 16–18 h post-insemination.

*Global *P* values were calculated using weighted Kruskal–Wallis tests for continuous variables and weighted chi-square tests for categorical variables.

†Pairwise comparisons were adjusted using the Holm method to control the family-wise error rate at α = 0.05. Holm-adjusted *P* values are reported for pairwise comparisons in this table.

Gn, gonadotropin; LH, luteinizing hormone; E_2_, estradiol; P, progesterone; 2PN, two-pronuclei; Ant, GnRH antagonist; Mild, mild stimulation; PPOS, progestin-primed ovarian stimulation.

**Table 2:** Weighted multivariable logistic regression analysis: pairwise comparisons of protocols for CLBR and CCPR.

Comparison	Cumulative live birth rate (CLBR) aOR (95% CI)	*P* value	Cumulative clinical pregnancy rate (CCPR) aOR (95% CI)	*P* value
Pairwise comparisons				
Mild stimulation vs. GnRH antagonist (reference)	0.83 (0.52–1.33)	0.448	0.86 (0.54–1.35)	0.508
PPOS vs. GnRH antagonist (reference)	1.15 (0.70–1.89)	0.579	1.08 (0.66–1.75)	0.767
PPOS vs. Mild stimulation (reference)	1.38 (0.85–2.24)	0.195	1.26 (0.78–2.02)	0.347
Model summary				
Hosmer-Lemeshow test P value		0.119		<0.001
Nagelkerke R²		0.067		0.063

Data are weighted estimates after entropy balancing.

All pairwise comparisons were derived from a single weighted multivariable logistic regression model for each outcome, with stimulation protocol (three categories), age (continuous), and AMH (continuous) as independent variables. Complete pairwise effect estimates were obtained by rotating the reference group.

Hosmer-Lemeshow test *P* > 0.05 indicates adequate model calibration.

Nagelkerke R² is a pseudo R-squared measure indicating the relative explanatory power of the model, useful for comparing models fitted to the same data.

aOR, adjusted odds ratio; CI, confidence interval; CLBR, cumulative live birth rate; CCPR, cumulative clinical pregnancy rate.

For the primary confirmatory endpoints (CLBR and CCPR), weighted multivariable logistic regression models were also constructed to estimate protocol-specific effects after additional adjustment for age and AMH. This adjustment was performed for two reasons: (1) although entropy balancing achieved near-perfect balance across all nine covariates, adjusting for the two strongest prognostic factors (age and AMH) in the regression model provides an additional layer of confounding control and enhances the precision of effect estimates; (2) including all nine covariates in the regression model would increase model complexity without substantially altering the effect estimates, given that balance was already achieved. Pairwise comparisons were obtained by rotating the reference group; no multiple comparison correction was applied as these comparisons derive from the same model and do not constitute independent tests. Model calibration was assessed using the Hosmer-Lemeshow test (*P* > 0.05 indicating adequate calibration), and Nagelkerke R² was used as a measure of relative explanatory power.

For interaction analysis (**Supplementary Table 2**), weighted multivariable logistic regression models were constructed including the product term of stimulation protocol (three categories) × POSEIDON subgroup (two categories: Group 3 vs Group 4), with additional adjustment for AMH, based on entropy balancing weights from the overall cohort. A two-sided *P* < 0.05 was considered statistically significant, except where otherwise specified.

## 3 Results

### 3.1 Study population and entropy balancing

A total of 592 patients meeting the inclusion criteria were enrolled between January 2017 and June 2023. The flow of participants is depicted in **Figure 2**. Patients were divided into three groups based on stimulation protocol: GnRH antagonist (n = 203), mild stimulation (n = 243), and PPOS (n = 146).

**Figure 2:**
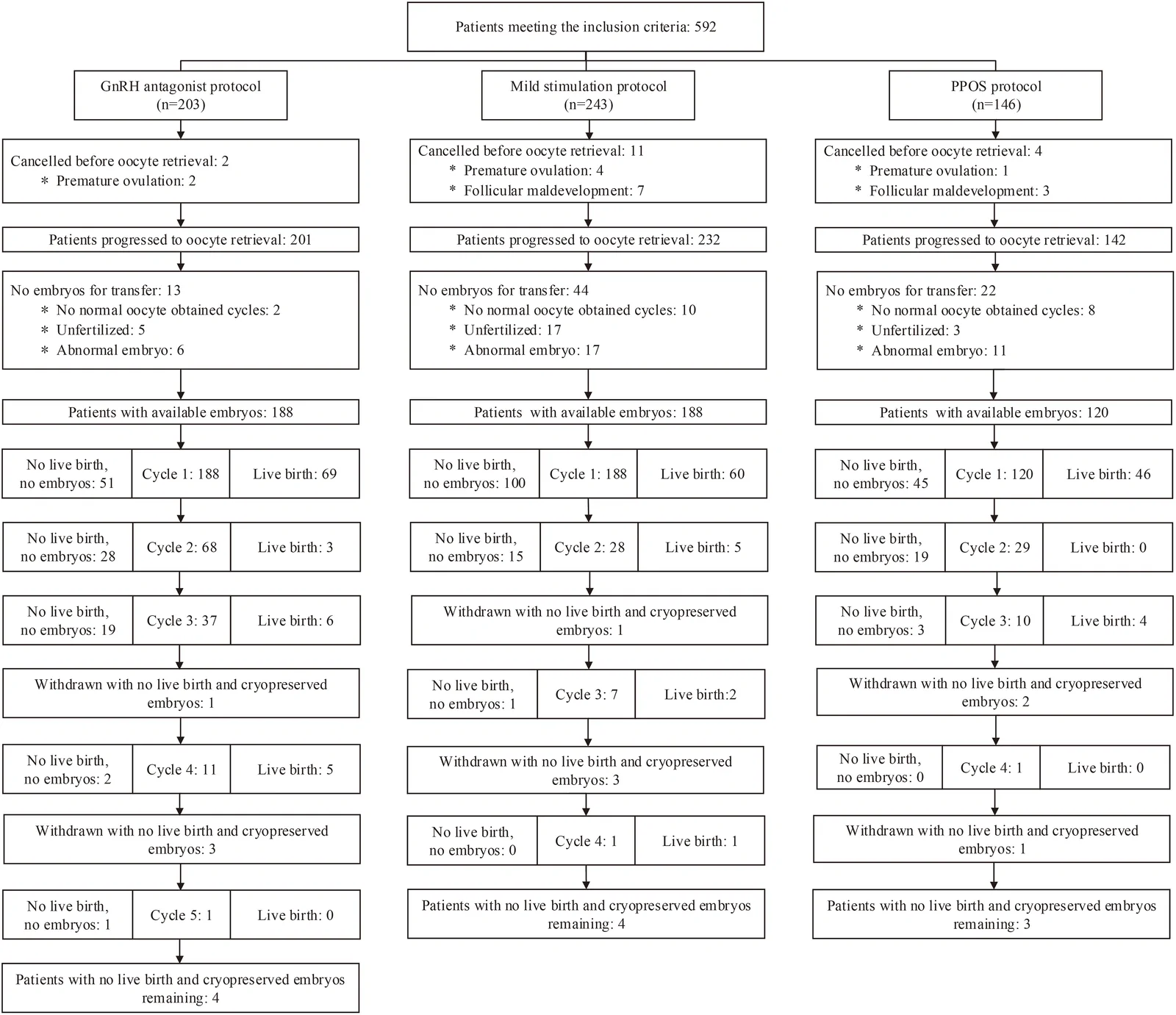
Flowchart of study participants.

Baseline characteristics before entropy balancing (**Table 3**) revealed notable inter-group differences across multiple covariates. Patients in the GnRH antagonist group were younger (median 34 years) with higher AMH (0.94 ng/mL) and AFC (6) compared to mild stimulation (age: 37 years; AMH: 0.64 ng/mL; AFC: 4) and PPOS (age: 36 years; AMH: 0.75 ng/mL; AFC: 4). Baseline FSH was lowest in the antagonist group (8.0 IU/L) compared to mild (9.7 IU/L) and PPOS (8.8 IU/L). Baseline LH, baseline E_2_, BMI, infertility duration, and infertility type showed Original SMD values ranging from 0.091 to 0.310, indicating relatively moderate to minor imbalances. The proportion of POSEIDON Group 3 patients was highest in the antagonist group (56.2%) compared to mild (38.3%) and PPOS (41.8%); because POSEIDON group is derived from age, AMH, and AFC, its SMD was not calculated. The largest imbalances were observed for AFC (Original SMD = 0.865), AMH (0.628), age (0.428), and baseline FSH (0.434), all substantially exceeding the 0.1 threshold, confirming the need for covariate balancing.

**Table 3:** Baseline characteristics before entropy balancing.

Variable	GnRH antagonist (n=203)	Mild stimulation (n=243)	PPOS (n=146)	Original SMD
Age (years)	34.0 (31.0–38.0)	37.0 (32.0–40.0)	36.0 (32.0–39.0)	0.428
AMH (ng/mL)	0.94 (0.73–1.10)	0.64 (0.36–0.90)	0.75 (0.40–0.99)	0.628
AFC	6 (4–9)	4 (3–5)	4 (3–6)	0.865
bFSH (IU/L)	8.0 (6.6–9.4)	9.7 (7.0–12.6)	8.8 (6.9–11.8)	0.434
bLH (IU/L)	4.8 (3.7–6.1)	5.0 (3.8–6.8)	5.1 (3.9–7.3)	0.310
bE_2_ (pmol/L)	151.2 (109.2–197.1)	156.2 (100.9–224.5)	150.2 (97.7–205.2)	0.150
BMI (kg/m²)	22.1 (20.4–24.4)	21.9 (20.1–24.2)	22.1 (20.6–24.1)	0.091
Infertility duration (years)	2.0 (1.0–4.0)	3.0 (1.0–5.0)	2.0 (1.0–4.0)	0.140
Infertility type				
Primary, n (%)	87 (42.9)	116 (47.7)	68 (46.6)	0.098
Secondary, n (%)	116 (57.1)	127 (52.3)	78 (53.4)	
POSEIDON group				
Group 3, n (%)	114 (56.2)	93 (38.3)	61 (41.8)	/
Group 4, n (%)	89 (43.8)	150 (61.7)	85 (58.2)	

Data are presented as median (interquartile range) or n (%).

SMD was calculated as the maximum absolute difference between any two of the three groups. SMD > 0.1 indicates imbalance.

AMH, anti-Müllerian hormone; AFC, antral follicle count; BMI, body mass index; FSH, follicle-stimulating hormone; LH, luteinizing hormone; E_2_, estradiol; POSEIDON, Patient-Oriented Strategies Encompassing Individualized Oocyte Number.

Entropy balancing successfully eliminated all baseline differences. After weighting (**Table 4**), all nine covariates achieved near-perfect balance with adjusted SMD values below 0.001. **Figure 1** provides a visual comparison of SMD before and after entropy balancing, demonstrating the substantial reduction in imbalance across all covariates. For example, the weighted median age across the three groups was 35.0, 36.0, and 36.0 years; weighted median AMH was 0.81, 0.74, and 0.76 ng/mL; and weighted median AFC was 5, 4, and 4. The distribution of POSEIDON groups after weighting was also balanced (Group 3: 44.8%, 45.1%, and 43.1%), confirming that the entropy balancing procedure successfully balanced the measured baseline covariates that could have confounded the comparison of protocol effects. Weight diagnostics indicated that the entropy balancing procedure preserved adequate effective sample size across all groups, with an overall Kish effective sample size of 448 (ESS/N = 0.76) (**Supplementary Table 1**).

**Table 4:** Baseline characteristics after entropy balancing.

Variable	GnRH antagonist (n=203)	Mild stimulation (n=243)	PPOS (n=146)	Adjusted SMD
Age (years)	35.0 (32.0–38.9)	36.0 (31.0–40.0)	36.0 (32.0–39.0)	0.000
AMH (ng/mL)	0.81 (0.52–1.04)	0.74 (0.48–0.98)	0.76 (0.45–1.03)	0.000
AFC	5 (3–7)	4 (3–6)	4 (3–7)	0.000
bFSH (IU/L)	8.6 (7.0–12.0)	9.3 (6.5–11.8)	8.5 (6.8–11.2)	0.000
bLH (IU/L)	5.0 (3.9–6.5)	4.9 (3.8–6.5)	4.8 (3.8–6.9)	0.000
bE_2_ (pmol/L)	145.1 (102.4–192.7)	156.1 (99.9–222.8)	151.7 (104.4–211.2)	0.000
BMI (kg/m²)	22.1 (20.4–24.1)	21.9 (20.1–24.1)	22.1 (20.7–24.3)	0.000
Infertility duration (years)	2.0 (1.0–4.1)	3.0 (1.0–4.0)	2.0 (1.0–4.0)	0.000
Infertility type				
Primary, %	45.8	45.8	45.8	0.000
Secondary, %	54.2	54.2	54.2	
POSEIDON group				
Group 3, %	44.8	45.1	43.1	/
Group 4, %	55.2	54.9	56.9	

Data are weighted estimates after entropy balancing.

Continuous variables are presented as weighted median (weighted interquartile range). Categorical variables are presented as weighted percentages (%).

SMD was calculated as the maximum absolute difference between any two of the three groups.

AMH, anti-Müllerian hormone; AFC, antral follicle count; BMI, body mass index; FSH, follicle-stimulating hormone; LH, luteinizing hormone; E_2_, estradiol; POSEIDON, Patient-Oriented Strategies Encompassing Individualized Oocyte Number.

### 3.2 Ovarian stimulation and embryology outcomes

Weighted ovarian stimulation and embryological outcomes are summarized in **Table 1**. Significant differences were observed across protocols in Gn consumption, hormonal profiles, follicular response, and embryo development.

Stimulation parameters. The mild stimulation protocol required significantly lower total Gn dose (median 1400 IU), shorter stimulation duration (10 days), and lower starting dose (150 IU) compared to GnRH antagonist (2089 IU, 10 days, 225 IU) and PPOS (2700 IU, 11 days, 225 IU) (all pairwise *P* < 0.001, except antagonist vs PPOS for Gn duration: Holm-adjusted *P* = 0.344).

Hormonal profiles. Trigger-day LH was significantly higher in mild stimulation (median 6.3 U/L) compared to antagonist (3.3 U/L) and PPOS (3.3 U/L) (global *P* < 0.001). E_2_ was highest in the antagonist group (4108 pmol/L), intermediate in mild (3919 pmol/L), and lowest in PPOS (2941 pmol/L) (global *P* < 0.001). Progesterone levels differed significantly (global *P* < 0.001), with PPOS showing the highest median (1.9 nmol/L), reflecting the exogenous progestin administration (medroxyprogesterone acetate) inherent to the PPOS protocol.

Follicular response and oocyte yield. The GnRH antagonist protocol demonstrated superior follicular response and oocyte yield, with higher total follicles (median 6), dominant follicles (4), oocytes retrieved (4), and MII oocytes (3) compared to mild stimulation (4, 3, 3, 2) (all pairwise *P* < 0.001). PPOS showed intermediate outcomes (4, 3, 4, 3).

Embryo development. The antagonist protocol achieved higher numbers of 2PN zygotes (median 3), usable embryos (2), and high-quality embryos (1) compared to mild stimulation (2, 1, 0) and PPOS (2, 2, 0) (all pairwise *P* < 0.001).

Adverse events. Despite the antagonist’s superior quantitative embryo yield, the rates of cycles with no oocytes retrieved (6.0% vs. 6.4% vs. 7.6%, global *P* = 0.888) and cycle cancellation due to no usable embryos (16.5% vs. 19.3% vs. 16.2%, global *P* = 0.786) were comparable across all three protocols, with all pairwise Holm-adjusted *P* values = 1.000.

For the binary outcome of obtaining at least one usable embryo, the weighted proportions were 83.5% for antagonist, 80.7% for mild, and 83.8% for PPOS (global P = 0.786). The corresponding unweighted event counts were: antagonist, 188/203; mild, 188/243; PPOS, 120/146. The risk differences based on the weighted proportions were: antagonist vs mild, 2.8% (95% CI: −4.3% to 9.9%); antagonist vs PPOS, −0.3% (95% CI: −8.1% to 7.5%); and PPOS vs mild, 3.1% (95% CI: −4.6% to 10.8%), all with confidence intervals crossing zero.

The premature LH surge rate was lowest in the PPOS group (1.5%), followed by antagonist (4.1%) and mild stimulation (8.6%) (global P = 0.047; Ant vs Mild: Holm-adjusted *P* = 0.470; Ant vs PPOS: Holm-adjusted *P* = 0.470; Mild vs PPOS: Holm-adjusted *P* = 0.005).

### 3.3 Cumulative live birth and clinical pregnancy rates

Weighted cumulative clinical outcomes are presented in **Table 5**. The CLBR per initiated cycle did not differ significantly among the three protocols (global *P* = 0.452): 32.8% (95% CI: 25.8–40.8%) for GnRH antagonist, 29.2% (95% CI: 23.4–35.8%) for mild stimulation, and 35.8% (95% CI: 28.2–44.2%) for PPOS. Holm-adjusted pairwise comparisons confirmed no significant differences (antagonist vs mild: *P* = 0.933; antagonist vs PPOS: *P* = 0.596; mild vs PPOS: *P* = 0.602). The corresponding absolute risk differences for CLBR were: antagonist vs mild, 3.6% (95% CI: −5.0% to 12.2%); antagonist vs PPOS, −3.0% (95% CI: −13.1% to 7.1%); and PPOS vs mild, 6.6% (95% CI: −3.0% to 16.2%), all with confidence intervals crossing zero. CCPR showed a similar pattern (global *P* = 0.649): 37.9% (95% CI: 30.4–46.1%) for antagonist, 34.6% (95% CI: 28.3–41.4%) for mild, and 39.5% (95% CI: 31.6–48.0%) for PPOS, with no significant pairwise differences (all Holm-adjusted *P* > 0.05).

**Table 5:** Weighted cumulative clinical outcomes per initiated cycle, with unweighted event counts.

Outcome	GnRH antagonist (n=203)	Mild stimulation (n=243)	PPOS (n=146)	*P* value*	Holm-adjusted *P* values†
Cumulative live birth rate (CLBR), % (95% CI) [unweighted n/N]	32.8 (25.8–40.8) [83/203]	29.2 (23.4–35.8) [68/243]	35.8 (28.2–44.2) [50/146]	0.452	Ant vs Mild: 0.933; Ant vs PPOS: 0.596; Mild vs PPOS: 0.602
Cumulative clinical pregnancy rate (CCPR), % (95% CI) [unweighted n/N]	37.9 (30.4–46.1) [94/203]	34.6 (28.3–41.4) [79/243]	39.5 (31.6–48.0) [55/146]	0.649	Ant vs Mild: 1.000; Ant vs PPOS: 0.784; Mild vs PPOS: 1.000
Patients with live birth and cryopreserved embryos remaining, % (95% CI) [unweighted n/N]	23.6 (17.6–30.9) [62/203]	17.6 (12.9–23.5) [38/243]	21.5 (15.3–29.4) [29/146]	0.405	Ant vs Mild: 0.476; Ant vs PPOS: 0.668; Mild vs PPOS: 0.754
Patients with no live birth but cryopreserved embryos remaining, % (95% CI) [unweighted n/N]	1.4 (0.5–3.9) [4/203]	2.8 (1.0–7.2) [4/243]	2.4 (0.8–7.0) [3/146]	0.690	Ant vs Mild: 1.000; Ant vs PPOS: 0.994; Mild vs PPOS: 0.857

Data are weighted estimates after entropy balancing.

Unweighted event counts (n/N) are shown in brackets for each group.

All rates are calculated per initiated cycle.

Percentages and 95% confidence intervals (CI) were estimated using the Wilson method with continuity correction.

*Global *P* values were calculated using weighted chi-square tests for categorical outcomes.

†Pairwise comparisons were adjusted using the Holm method to control the family-wise error rate at α = 0.05.

Ant, GnRH antagonist; Mild, mild stimulation; PPOS, progestin-primed ovarian stimulation; CLBR, cumulative live birth rate; CCPR, cumulative clinical pregnancy rate.

The proportion of patients with no live birth but cryopreserved embryos remaining was low and comparable across groups (1.4%, 2.8%, 2.4%; global *P* = 0.690). The proportion achieving live birth with remaining cryopreserved embryos was 23.6%, 17.6%, and 21.5% for antagonist, mild, and PPOS, respectively (global *P* = 0.405), with no significant pairwise differences after Holm adjustment.

Weighted multivariable logistic regression (**Table 2**) confirmed that protocol was not an independent predictor of CLBR after adjustment for age and AMH. Compared with antagonist, mild stimulation showed aOR = 0.83 (95% CI: 0.52–1.33; *P* = 0.448), and PPOS showed aOR = 1.15 (95% CI: 0.70–1.89; *P* = 0.579). Direct comparison between PPOS and mild showed aOR = 1.38 (95% CI: 0.85–2.24; *P* = 0.195). The CLBR model demonstrated good calibration (Hosmer-Lemeshow *P* = 0.119) with Nagelkerke R² = 0.067. For CCPR, no significant differences were observed (mild vs antagonist: aOR = 0.86, 95% CI: 0.54–1.35, *P* = 0.508; PPOS vs antagonist: aOR = 1.08, 95% CI: 0.66–1.75, *P* = 0.767; PPOS vs mild: aOR = 1.26, 95% CI: 0.78–2.02, *P* = 0.347). The CCPR model showed Hosmer-Lemeshow *P* < 0.001 and Nagelkerke R² = 0.063; the significant Hosmer-Lemeshow test suggests suboptimal calibration, and therefore the CCPR results should be interpreted with caution.

**Supplementary Table 2** presents the interaction analysis between stimulation protocol and POSEIDON subgroup. The interaction term was not significant for CLBR (*P* for interaction = 0.384) or CCPR (*P* for interaction = 0.213), indicating consistent treatment effects across POSEIDON groups 3 and 4. The Hosmer-Lemeshow test for both interaction models was significant (*P* < 0.001), suggesting suboptimal calibration; these results should therefore be interpreted with caution.

Safety outcomes. No cases of moderate or severe OHSS occurred in any of the three groups. Miscarriage rates were 9.6% (9/94) for antagonist, 15.2% (12/79) for mild, and 14.5% (8/55) for PPOS. Multiple pregnancy rates were 14.9% (14/94), 19.0% (15/79), and 9.1% (5/55), respectively. Ectopic pregnancy occurred in two patients in the antagonist group (2.1%, 2/94) and in none in the other two groups.

## 4 Discussion

This study provides a rigorous, head-to-head comparison of three ovarian stimulation protocols in a homogeneous cohort of POSEIDON Groups 3 and 4 patients, yielding several findings with direct clinical implications.

### 4.1 Primary finding: no statistically significant differences in cumulative live birth or clinical pregnancy rates

The principal finding of our study is that, after rigorous adjustment for baseline differences, the three protocols showed no statistically significant differences in CLBR and CCPR in POSEIDON Groups 3 and 4 patients. The weighted CLBR ranged from 29.2% to 35.8%, with no significant differences between any two protocols (global *P* = 0.452). The weighted CCPR showed a similar pattern: 37.9%, 34.6%, and 39.5% for the antagonist, mild, and PPOS groups, respectively (global *P* = 0.649). These findings were robust to multivariable adjustment for age and AMH (**Table 2**), confirming that protocol choice was not independently associated with the ultimate chance of live birth or clinical pregnancy.

Several observations support the validity of this conclusion. The proportion of patients with no live birth but remaining cryopreserved embryos was low and balanced across groups (1.4–2.8%), indicating complete follow-up and minimizing the risk of biased CLBR estimates. The consistency between unadjusted weighted comparisons and multivariable-adjusted estimates further strengthens confidence in the null finding.

Our results align with previous studies demonstrating comparable CLBR between alternative and conventional protocols in low-prognosis patients (13, 19, 21, 30), but contrast with reports suggesting superior outcomes with GnRH antagonists (31, 32). This discrepancy likely reflects several key differences between the studies. First, patient selection varied substantially across studies. Most previous comparative studies included heterogeneous populations with mixed infertility etiologies such as tubal factors, male factor infertility, and endometriosis (19, 20), whereas our study restricted inclusion to patients whose sole indication for IVF was meeting POSEIDON criteria, thereby minimizing confounding from other etiologies. Second, the use of cumulative live birth rate as the primary outcome distinguishes our study from earlier work that often relied on per-cycle or per-transfer outcomes. CLBR is inherently more informative for poor-prognosis patients who typically require multiple transfers, and may therefore reveal different treatment effects than per-cycle analyses. Third, our statistical approach—entropy balancing—offers a more rigorous adjustment for baseline confounding than conventional multivariable regression or propensity score matching, and may account for some of the discrepancies between our findings and previous reports. Fourth, the recent three-way comparison by Kasliwal et al. (25) reported superior blastocyst yield with PPOS in women aged 21–35 years, but that study did not assess cumulative live birth; importantly, their embryological findings do not contradict our clinical observations. The apparent inconsistency underscores the distinction between embryological productivity and cumulative clinical success, particularly in patients with severely diminished ovarian reserve.

The interaction analysis (**Supplementary Table 2**) revealed no significant effect modification by POSEIDON subgroup for either CLBR (*P* for interaction = 0.384) or CCPR (*P* for interaction = 0.213), indicating consistent treatment effects across younger (Group 3) and older (Group 4) patients. This is clinically reassuring, as it suggests that age alone should not drive protocol selection within this population.

### 4.2 Embryological superiority without clinical translation: a critical insight

The GnRH antagonist protocol demonstrated consistently superior embryological outcomes: more oocytes retrieved, usable embryos, and high-quality embryos. However, this advantage did not translate into higher CLBR or CCPR. This dissociation demonstrates that in the POSEIDON 3/4 population—a group with inherently limited ovarian reserve—the quantitative advantage in embryo yield from more intensive stimulation does not necessarily confer a proportionate advantage in cumulative live birth.

This dissociation may reflect several interrelated factors. First, in patients with diminished ovarian reserve, more intensive stimulation may recruit a broader cohort of follicles, but the additional follicles recruited beyond the optimal range may be of lower intrinsic quality, with higher rates of meiotic abnormalities or developmental incompetence, diluting the cumulative benefit of having more embryos. Second, the rate-limiting step for achieving live birth in this population may be the intrinsic reproductive potential of each patient—determined by age and ovarian reserve—rather than the number of embryos available.

Third, an exploratory finding observed in our data—which we tentatively term a potential threshold effect—provides supportive evidence for this interpretation. As shown in **Table 1**, while the antagonist protocol yielded significantly more usable and high-quality embryos, the rates of cycles with no oocytes retrieved and cycle cancellation due to no usable embryos were virtually identical across all three protocols. This means that the choice of stimulation protocol did not affect whether a patient would obtain at least one usable embryo—the critical threshold for proceeding to embryo transfer. Among patients who crossed this threshold, the antagonist protocol produced more embryos, but the additional embryos did not increase the cumulative probability of live birth. This suggests that in this population, once a patient has at least one embryo, the number of additional embryos has diminishing marginal returns.

If confirmed in future studies, this potential threshold effect would have important clinical implications. For patients with DOR, the most anxiety-provoking question is often not “how many embryos will I get?” but “will I get any embryos at all?” Our data provide reassurance that the choice between the three protocols does not significantly influence the risk of cycle cancellation due to no usable embryos—the rates were 16.5%, 19.3%, and 16.2%, respectively, statistically indistinguishable. This empowers clinicians to select a protocol based on other considerations (patient preference, cost, convenience) without substantially increasing the patient’s risk of “walking away empty-handed.”

We acknowledge that this threshold effect is exploratory. While the rates of cycle cancellation due to no usable embryos were statistically indistinguishable across protocols (16.5%, 19.3%, and 16.2%; global P = 0.786), these findings do not establish causality. The observation that protocol choice was not a significant predictor of obtaining at least one usable embryo in our adjusted analyses may reflect the dominant influence of baseline ovarian reserve parameters (AMH and AFC) over stimulation protocol in this population. However, confirmation of this exploratory finding requires prospective studies specifically designed to test the threshold hypothesis, with rigorous control for confounding.

### 4.3 Protocol-specific characteristics: informing individualized choice

While no statistically significant difference in CLBR was observed, the three protocols demonstrated distinctly different profiles that can guide individualized selection.

GnRH antagonist protocol offers the highest embryo yield, making it the preferred strategy for patients who prioritize maximizing the number of embryos, particularly those desiring more than one child. However, this comes at the cost of higher Gn consumption and greater medication burden. The numerically higher proportion of patients with remaining cryopreserved embryos (23.6% vs. 17.6% and 21.5%), although not statistically significant, may still represent a clinically meaningful advantage for patients seeking future sibling pregnancies.

PPOS protocol emerges as a compelling alternative, offering several unique advantages. Most notably, it demonstrated the lowest premature LH surge rate (1.5%) among the three protocols—lower than even the GnRH antagonist (4.1%). This finding challenges the conventional assumption that GnRH antagonists are superior for LH surge suppression and supports the growing evidence that progestin priming is highly effective (15–18). PPOS also offers the convenience of oral administration, eliminating the need for daily injections. For patients who prioritize convenience and simplicity without compromising the ultimate chance of live birth, PPOS represents an excellent option.

Mild stimulation protocol, despite higher premature LH surge rate (8.6%) and numerically higher cycle cancellation (19.3%), remains a valid option for patients seeking minimal intervention and reduced medication burden. The significantly lower Gn requirement translates to substantially lower medication costs and potentially fewer side effects. Importantly, the higher LH surge rate did not translate into inferior CLBR, suggesting that with appropriate monitoring, mild stimulation can achieve comparable cumulative success.

### 4.4 Strengths and limitations

Several methodological features enhance the credibility of our findings. We employed entropy balancing, a covariate balancing weighting method that achieves exact covariate balance without relying on model specification or discarding unmatched observations. This offers several advantages over conventional approaches: unlike multivariable regression, it does not assume linear relationships; unlike propensity score matching, it retains the full sample; and unlike inverse probability weighting, it does not depend on correctly specified treatment models. Furthermore, entropy balancing naturally accommodates multi-group comparisons, a feature particularly valuable for our three-protocol comparison. After entropy balancing, all nine prespecified covariates achieved near-perfect balance (SMD < 0.001).

We also restricted our analysis to first IVF cycles. This design choice eliminates the confounding influence of prior cycle outcomes on protocol selection—a critical advantage that ensures protocol assignment is not influenced by previous treatment results. In patients with prior IVF history, protocol assignment for subsequent cycles is often influenced by previous outcomes, creating a confounding feedback loop. By restricting to first cycles, we ensured that protocol selection was based solely on baseline characteristics, providing a cleaner comparison.

Our strict inclusion criteria—restricting to patients whose sole indication for IVF was meeting the POSEIDON Groups 3 or 4 criteria—produced a highly homogeneous cohort of anticipated poor responders, eliminating potential confounders such as tubal factors, male factor infertility, and endometriosis. This design choice ensures that our findings are directly relevant to the clinical question of which stimulation protocol best serves patients with diminished ovarian reserve in the absence of other infertility diagnoses.

The complete follow-up, evidenced by the low and balanced rates of patients with no live birth but cryopreserved embryos remaining (1.4% to 2.8%), further strengthens the validity of our CLBR estimates.

We acknowledge the inherent limitations of the retrospective design. The single-center nature of this study may limit generalizability, but it also reduces between-center heterogeneity in clinical protocols, laboratory conditions, and embryologist expertise, enhancing internal validity. Furthermore, embryo quality was assessed microscopically based on morphological criteria, as preimplantation genetic testing (PGT) was not routinely performed for this population in our clinical practice during the study period; therefore, embryonic chromosomal status remains unknown. Additionally, although we excluded severe uterine pathologies (e.g., adenomyosis, submucous fibroids) in our inclusion criteria, specific endometrial factors affecting endometrial receptivity were not systematically studied or excluded, which may influence IVF outcomes. These limitations should be addressed in future prospective studies. Additionally, because the study period spanned 2017 to 2023, residual confounding from temporal changes in laboratory or transfer practices cannot be fully excluded. Our sample size, while substantial, may still be underpowered to detect small differences in rare outcomes. We did not evaluate economic factors or patient preferences, which are increasingly important in clinical decision-making (33, 34). Finally, the significant Hosmer-Lemeshow test for the CCPR model suggests suboptimal calibration, and these results should be interpreted with caution. Neonatal outcomes were not systematically collected in this retrospective study.

### 4.5 Conclusions

In POSEIDON Groups 3 and 4 patients undergoing their first IVF cycle, after rigorous adjustment for baseline differences, the GnRH antagonist, mild stimulation, and PPOS protocols showed no statistically significant differences in cumulative live birth and clinical pregnancy rates, despite significant variations in intermediate embryological outcomes. Critically, while the antagonist protocol produces more usable and high-quality embryos, it does not reduce the risk of cycle cancellation due to no usable embryos—all three protocols carry a similar probability of yielding at least one embryo. This exploratory finding suggests that in this population, the ability to obtain any embryo may be more strongly associated with intrinsic patient characteristics than with the choice of stimulation protocol. These findings empower individualized protocol selection based on patient priorities and clinical context, moving beyond a one-size-fits-all approach.

For patients who prioritize maximizing embryo yield, the GnRH antagonist protocol may be a preferred strategy, as it offers the highest likelihood of generating surplus embryos for future sibling projects. However, this should be weighed against higher gonadotropin requirements and greater medication burden. For patients who seek convenience and simplicity, the PPOS protocol may be a suitable alternative, offering oral administration and the lowest premature LH surge rate among the three protocols, with no statistically significant difference in CLBR. For patients who prioritize minimal intervention, the mild stimulation protocol may be a valid option, achieving comparable CLBR with substantially lower medication requirements. However, these considerations are speculative, as cost, burden, convenience, and patient preferences were not directly measured in this study. Future prospective, multi-center studies incorporating formal cost-effectiveness analyses and patient-reported outcome measures are warranted to validate and refine these personalized treatment strategies for this challenging population.

## Data Availability

The original contributions presented in the study are included in the article/**Supplementary Material**. Further inquiries can be directed to the corresponding author.
