# Supplementary material for: Comparative effectiveness of GnRH antagonist, mild stimulation, and PPOS protocols in POSEIDON groups 3 and 4: a retrospective cohort study with entropy balancing
Source: Front Endocrinol (Lausanne). 2026 Oct 21;17:1969038. doi: 10.3389/fendo.2026.1969038 (PMC13730927; doi:10.3389/fendo.2026.1969038)
Supplement: Supplementary file 1 [file Table1.docx]

Supplementary Material

**Supplementary Table 1.** Entropy balancing weight diagnostics by treatment group

| Statistic | Antagonist (n = 203) | Mild (n = 243) | PPOS (n = 146) |
| --- | --- | --- | --- |
| Weight: Min | 0.308 | 1.030 | 1.170 |
| Weight: Q1 | 1.590 | 1.790 | 3.520 |
| Weight: Median | 2.260 | 2.180 | 4.000 |
| Weight: Q3 | 3.380 | 2.800 | 4.490 |
| Weight: Max | 13.700 | 14.300 | 7.030 |
| Weight: Mean | 2.920 | 2.440 | 4.050 |
| Weight: SD | 2.230 | 1.170 | 0.972 |
| Coefficient of variation (CV) | 0.765 | 0.482 | 0.240 |
| Kish effective sample size (ESS) | 128 | 197 | 138 |

Notes:
Weight statistics are derived from the entropy balancing procedure implemented using the ebal method in the WeightIt package (version 1.7.0) in R (version 4.5.1). The estimand was the average treatment effect (ATE), and only first moments were balanced (moments = 1). No quantile constraints, interaction terms, or weight truncation were applied. Weights were normalized such that their overall mean equaled 3 (i.e., the number of treatment groups). The overall Kish effective sample size was 448, corresponding to an ESS/N ratio of 0.76, indicating that 76% of the original sample size was retained after weighting.

Abbreviations: Q1, first quartile (25th percentile); Q3, third quartile (75th percentile); SD, standard deviation; CV, coefficient of variation; ESS, effective sample size; ATE, average treatment effect.

**Supplementary Table 2.** Interaction analysis of stimulation protocol and POSEIDON subgroups for CLBR and CCPR

| Outcome | *P* for interaction¹ | Hosmer-Lemeshow test  *P* value | Nagelkerke R² |
| --- | --- | --- | --- |
| Cumulative live birth rate (CLBR) | 0.384 | <0.001 | 0.073 |
| Cumulative clinical pregnancy rate (CCPR) | 0.213 | <0.001 | 0.069 |

Notes:

¹*P* for interaction was derived from weighted multivariable logistic regression models including the product term of stimulation protocol (three categories) × POSEIDON subgroup (two categories: Group 3 vs Group 4), with additional adjustment for AMH, based on entropy balancing weights from the overall cohort.

Given the exploratory nature of these analyses, the significance threshold was set at α = 0.10.

Hosmer-Lemeshow test *P* > 0.05 suggests adequate model calibration.

Nagelkerke R² is a pseudo R-squared measure indicating the relative explanatory power of the model, useful for comparing models fitted to the same data.

Abbreviations: CLBR, cumulative live birth rate; CCPR, cumulative clinical pregnancy rate; POSEIDON, Patient-Oriented Strategies Encompassing Individualized Oocyte Number; AMH, anti-Müllerian hormone.
